# Non-linear effects of technological immersion on directed attention restoration: an exploratory study across smartphone, tablet, and virtual reality interfaces

**DOI:** 10.3389/fpsyg.2026.1942295

**Published:** 2026-08-31

**Authors:** Jean-Christophe Sakdavong, Jason Melliere, Mouhammadou Dia, Anaïs Niveau, Carole Brunet, Sylvain Pleuchot, Samuel Désirée

**Affiliations:** Laboratoire CLLE (UMR5263), University of Toulouse, Toulouse, France

**Keywords:** Attention Restoration Theory, Bayesian inference, biophilic interventions, mental fatigue, SART, technological immersion

## Abstract

**Background:**

While Virtual Reality (VR) is increasingly adopted for digital biophilic interventions aimed at mitigating mental fatigue, it remains unclear whether higher technological immersion linearly enhances directed attention restoration.

**Objectives:**

This study investigated the restorative effects of three levels of immersion: smartphone (low), tablet (moderate) and VR headset (high), on directed attention following exposure to a nature video.

**Methods:**

Ninety adults completed the Sustained Attention to Response Task (SART) before and after the intervention.

**Results:**

While intra-group analyses provided extreme evidence for attentional restoration using a tablet (*p* < 0.001, *BF*_10_ = 6573) and strong evidence for VR (*p* = 0.003, *BF*_10_ = 12.7), the overall inter-group differentiation failed to reach strict frequentist significance (*p* = 0.085). However, Bayesian model comparisons revealed a non-monotonic pattern of efficacy. Crucially, while a Bayesian Repeated Measures ANOVA showed only anecdotal evidence (*BF*_*inclusion*_ = 2.33) for a Time × Device interaction, *post-hoc* comparisons yielded moderate evidence for the null hypothesis when comparing the smartphone to the VR headset (*BF*_10_ = 0.215).

**Conclusions:**

These data indicate that the highest level of immersion provided no restorative advantage over the lowest level, potentially due to the cognitive overhead associated with VR. These findings provide moderate evidence against a linear relationship between immersion and restoration, highlighting the need to carefully consider the biomechanical and cognitive confounds of immersive technologies.

## Introduction

The rapid urbanization of modern society has drastically curtailed daily access to natural environments. As urban sprawl replaces green spaces with artificial infrastructure, individuals are increasingly exposed to chronic environmental stressors (such as traffic noise, congestion, and visual clutter) that accelerate attentional fatigue (Barton and Pretty, 2010; Linnell and Caparos, 2020).

Simultaneously, individuals are exposed to continuous cognitive demands, including work-related responsibilities, digital interactions, and the pressures of modern lifestyles. These persistent cognitive loads frequently rely on directed attention, a finite resource that, when overused, can lead to attentional fatigue (Moran, 2019). Research has demonstrated that urbanization alters attentional selection processes, leading to increased cognitive load and reduced engagement in focused tasks (Linnell et al., 2013). Furthermore, individuals in urban settings show higher neural vigilance and lower restoration of attention, as evidenced by fMRI studies on urban green infrastructure (Jiang et al., 2023). Exposure to nature has been increasingly explored as a method to mitigate attentional fatigue (Varkovetski, 2016; van Oordt et al., 2023).

These biophilic interventions are primarily grounded in two complementary frameworks: Stress Recovery Theory (SRT), which emphasizes the affective and physiological modulation of stress (Ulrich, 1983), and Attention Restoration Theory (ART; Kaplan and Kaplan, 1989). While SRT focuses on emotional regulation, ART posits that natural settings provide an optimal balance of fascination, compatibility, extent, and the feeling of being away, all of which contribute to cognitive recovery. Because urban environments uniquely deplete directed attention, this study specifically focuses on the ART mechanisms of fatigue mitigation. However, in highly urbanized environments, direct exposure to nature is often impractical. To address this limitation, researchers have investigated artificial nature simulations, such as images, videos, or even virtual reality, as potential substitutes for real-world nature. These technologies offer immersive experiences that may replicate the cognitive benefits of direct nature exposure (Li et al., 2021).

A critical determinant of the restorative efficacy of these simulations appears to be the degree of immersion, which effectively acts as a proxy for the intensity of the biophilic dose. While VR has demonstrated superior restorative potential compared to standard screens in some contexts (Dillon and Cai, 2022), a significant gap remains in understanding how the intensity of technological immersion modulates these effects across the full spectrum of digital biophilic interventions. Specifically, it is unclear whether the dose-response relationship between immersion and restoration is linear: does increasing screen size and sensory isolation consistently enhance cognitive recovery, or are there diminishing returns due to cognitive overhead? This study addresses this gap by systematically investigating the dose-response effects of three distinct levels of immersion intensity on directed attention restoration.

### Attention Restoration Theory

#### Defining attention

Attention has been extensively studied over the years and among many different fields. While several models have been developed and updated or discarded over time, it can effectively be summed up as a process that allows for the efficient allocation of cognitive resources, ensuring that individuals can concentrate on tasks, interpret complex information, respond appropriately to their environment, and selectively focus on relevant stimuli while filtering out extraneous information (Mancas, 2016). James (1890) was among the first to distinguish between two primary forms of attention: Involuntary (or bottom-up) attention and voluntary (or top-down) attention.

Involuntary attention operates automatically, driven by external stimuli that capture cognitive resources without conscious effort (Baluch and Itti, 2011). It allows individuals to rapidly detect and respond to salient environmental cues such as sudden sounds, bright colors, or unexpected movements. Unlike voluntary attention, which requires effortful engagement and goal-directed control, involuntary attention is stimulus-driven and typically reflexive (Eimer et al., 1996). For example, one might see or hear a fight breaking out almost immediately in a crowded street despite not initially paying attention to the particular people involved. Involuntary attention is mediated by distinct neural pathways from those involved in voluntary attention. Event-related potentials research has shown that involuntary attentional shifts improve early sensory processing in the extrastriate cortex. These shifts happen about 130 ms after the stimulus starts (Fu et al., 2005). This modulation of sensory processing ensures that unexpected but potentially significant stimuli receive prioritized cognitive resources, a useful feature for threat detection and exploratory behavior.

Voluntary attention describes one's conscious focus on selected information, often while keeping irrelevant information and stimuli out of focus. This top-down attentional control is driven by internal cognitive processes rather than external sensory stimuli (Baluch and Itti, 2011). It enables individuals to allocate cognitive resources to specific tasks based on goals and expectations, an effortful mechanism that demands engagement from higher-order executive networks in the brain (Norman and Shallice, 1986; Petersen and Posner, 2012). Topdown attention can be subdivided into executive attention and sustained attention (Barton et al., 2020). Executive attention encompasses cognitive control mechanisms that allow for the selective prioritization of relevant stimuli while filtering out competing distractions (Diamond, 2013; Rueda et al., 2015). This function is critical in demanding cognitive tasks, where successful performance hinges on the ability to inhibit irrelevant information and adjust attentional focus dynamically (Petersen and Posner, 2012; Diamond, 2013).

Additionally, sustained attention refers to the ability to maintain cognitive engagement over an extended period, an essential skill for tasks that require prolonged focus without frequent attentional shifts (Rueda et al., 2015).

However, the consumption of attentional resources is not solely dictated by visual processing but is also deeply rooted in the biomechanical demands of device interaction. Recent research in embodied cognition suggests that the “active holding” of a mobile device constitutes a secondary motor task that competes for executive resources (Canning, 2005; Nagamatsu et al., 2011). Maintaining a static limb position while stabilizing a handheld device requires continuous proprioceptive monitoring and postural regulation, a process that is cognitively taxing and susceptible to dual-task interference (Doumas et al., 2009). Consequently, the physical act of holding a smartphone may impose an intrinsic “cognitive tax”, an unrecognized load that drains the very attentional reserves the intervention aims to restore. This contrasts with hands-free modalities, such as viewing a propped tablet or wearing a VR headset, where motor demands are significantly reduced.

#### Consequences of attentional fatigue

Maintaining top-down attention becomes increasingly challenging in environments filled with distractions, such as competing visual or auditory stimuli (Wilmer et al., 2017). While bottom-up attention does not fatigue, top-down attention is a finite resource that can be depleted, leading to mental fatigue (Moran, 2019). Cognitive function declines when voluntary attention is exhausted, making it more challenging to filter out irrelevant stimuli and continue to be productive (Hartig et al., 1991). This condition leads to inefficiencies in problem-solving and decision-making (Kaplan, 1995). Beyond these cognitive impairments, the depletion of attention has been linked to heightened stress levels, irritability, and lower cooperation (Cohen and Spacapan, 1978).

#### Restoring attention: the ART

Given the many negative effects of attentional fatigue, identifying effective strategies for restoring cognitive function is of paramount importance. Attention Restoration Theory, developed by Kaplan and Kaplan (1989), posits that prolonged engagement with voluntary attention in cognitively demanding tasks depletes attentional resources, leading to mental fatigue and decreased cognitive performance. However, exposure to specific environments, particularly those that engage attention effortlessly, can facilitate recovery by allowing the directed attention system to rest and replenish itself (Kaplan, 1995; Stevenson et al., 2018). Natural environments are particularly well-suited for this function, as they offer stimuli that capture attention involuntarily, allowing sustained focus to recover (Basu et al., 2019). In contrast, urban settings, filled with competing stimuli that require active filtering, tend to further tax the attentional system, exacerbating cognitive fatigue rather than alleviating it (Kaplan and Kaplan, 1989). The ART identifies four key attributes that define a restorative environment: fascination, extent, being away, and compatibility. These elements collectively determine how effectively an environment facilitates attentional recovery.

Fascination refers to the ability of an environment to capture attention effortlessly, reducing the need for effortful cognitive engagement. Kaplan (1995) described this type of fascination as a passive yet engaging form of attention that does not require conscious effort, allowing mental resources to be restored. Within this framework, Basu et al. (2019) argue that it is necessary to distinguish between the concepts of hard and soft fascination as they have different implications in terms of mental bandwidth. While fascination as a state implies little mental effort needed to focus, it is still important to distinguish between fascination with high mental resources available for self-reflection (soft fascination), such as seeing animals or flowing water, and fascination with low mental resources (hard fascination), such as action-packed media or urban distractions, which are engaging but cognitively taxing and leave little room for internal reflection. Optimal restorative settings involve soft fascination as they allow for a reprieve for attentional resources and leave room for “mental housekeeping” (Kaplan, 1993).

Extent describes the degree to which an environment provides a coherent and complex enough experience. Environments that offer depth and complexity, such as forests, vast plains, and expansive landscapes, create a sense of immersion that fosters restoration and naturally occupies the mind; however, extent can also come from the implied continuity of what we see and is thus not merely physical (Kaplan and Kaplan, 1989). As such, Herzog et al. (2003) rightfully point out that even an objectively small setting can have sufficient extent if it is complex enough to properly occupy the mind, citing Japanese gardens as an excellent example.

Being away refers to the psychological and physical separation from stress-inducing environments. A restorative setting allows individuals to disconnect from cognitively demanding tasks and everyday pressures. This detachment can occur physically, such as visiting a natural park, or psychologically, through an engaging simulated environment that removes individuals from their usual context of stress. For example, university students with access to nature views from a window experience higher psychological wellbeing partly due to the restorative effects of being away (Yusli et al., 2021).

Finally, compatibility describes the alignment between the environment and the individual's needs, preferences and behavioral inclinations. A setting is most restorative when it supports the desired goals and inclinations of the individual.

#### Individual connection to nature

The extent to which a person feels emotionally and psychologically connected to nature affects how they respond to its exposure. While individuals tend to naturally perceive nature as inherently restorative (Çelebi and Ayva, 2017), those with a stronger connection to nature are more likely to experience a heightened sense of restoration. Berto et al. (2018) examined how an individual's connection to nature moderates perceived restorativeness in various environmental settings. The study found that participants with a strong nature connection consistently rated natural environments as more restorative than those with lower connection levels. The authors cite Wilson, 1986 biophilia hypothesis (1986), which posits that humans evolved to possess an inherent affinity for nature, as an explanation for the fact that high-biophilic environments provided greater restorative benefits.

However, the relationship between nature connectedness and attentional restoration is not entirely straightforward. While studies indicate a positive correlation between perceived restorativeness and nature connectedness, objective cognitive performance outcomes do not always align with subjective perceptions. Johnson et al. (2022) found that individuals with high connectedness to nature reported feeling more restored after viewing images of nature; however, their sustained attention performance showed no significant improvement. This dissociation between subjective and objective restoration suggests that while an emotional bond with nature may enhance perceived benefits, it does not necessarily translate into measurable cognitive recovery.

### Immersion

#### Defining immersion

Immersion often refers to the level of physical or psychological submergence within a virtual space, relative to an individual's awareness of the real-world environment (Emma-Ogbangwo et al., 2014). Immersion is also frequently conceptualized as the objective level of sensory fidelity provided by a system. Following Slater's (2009) framework, we distinguish between technological immersion (defined by objective system properties such as field of view (FOV), resolution, and stereoscopy) and presence, the subjective psychological illusion of “being there” (Biocca, 1997). Crucially, this distinction is reinforced by meta-analytic evidence indicating that while objective immersive features, such as stereoscopic vision and wider fields of view, are significant predictors of presence, they exhibit only a medium effect size (*r* ≈ 0.39) (Cummings and Bailenson, 2016). This suggests that high technological immersion facilitates the illusion of place but does not strictly dictate the subjective quality of the experience, leaving room for other variables such as cognitive load or comfort to modulate the outcome. While Queiroz et al. (2018) describe this subjective state as “psychological immersion,” strictly differentiating the hardware's capabilities from the user's experience is critical for our analysis. Indeed, while high technological immersion is a prerequisite for presence, it does not guarantee positive cognitive outcomes. If the immersive hardware imposes an extraneous cognitive load (due to novelty, interface complexity, or physical discomfort) it may trigger high arousal rather than the relaxation required for ART (Makransky et al., 2021). Thus, the relationship between objective immersion and restoration may be constrained by the user's capacity to process the environment without cognitive overload.

#### Levels of immersion and attention restoration

Immersive virtual nature experiences have been shown to provide some of the benefits associated with real nature exposure (Jo et al., 2019; Li et al., 2021) and are generally considered a satisfactory replacement for real life when it comes to attentional restoration purposes. Still, real-life exposure to nature is consistently better than its replacements (Browning et al., 2020).

There appears to be a clear hierarchy among these replacements based on how immersive they are. For example, Valtchanov et al. (2010) showed that people who actively explored a virtual forest in VR felt happier and less stressed than people who watched a simple slideshow as a control. Dillon and Cai (2022) directly compared VR-based nature exposure to viewing nature on a computer screen and found that VR produced superior attention restoration effects. It seems logical to conclude that the intrinsically higher immersion that VR provides has concrete benefits in nature-based interventions compared to traditional two-dimensional media.

But what are the differences between these various traditional media? Sponselee et al. (2004) explored the effect of a small (31”) vs. large (72”) screen size on physiological stress reduction and affect when watching nature scenes, finding that larger screens enhanced skin conductance recovery, an indicator of stress reduction. However, self-reported affect did not significantly differ between conditions. Similar results were reported by de Kort et al. (2006), using the same screen sizes to manipulate immersion in a projected nature environment. They found that viewing a nature scene from the larger screen led to stronger physiological restoration from stress, as measured from heart period and skin conductance level, but subjective measures of self-reported affect were not significantly different across conditions.

Although the literature lacks other direct comparisons between different levels of immersion, such as smaller smartphone-sized screens and medium-sized computer screens or even tablets in the context of attentional restoration, we can still reasonably infer that larger screen sizes will consistently enhance restoration: Rigby et al. (2016) manipulated screen sizes and showed that a 30” monitor provided a greater sense of presence than a 13” laptop when watching movies. Generally speaking, larger screens consistently enhance presence, whether in video games or more passive forms of entertainment such as watching movies or videos (Hou et al., 2012; IJsselsteijn et al., 2001; Lombard et al., 2000). Therefore, based on these traditional media frameworks, it is logical to initially posit a linear model where attentional restoration increases alongside technological immersion (smartphone, tablet, and VR headset). However, this linear assumption remains to be tested against the potential cognitive overhead inherent to highly immersive systems.

If watching a nature video on a smartphone is shown to produce measurable attention restoration, this could open up practical everyday applications for cognitive recovery in situations where larger, more immersive devices are unavailable. At the same time, identifying how much additional benefit is gained as we move up the immersion scale can help determine whether increasingly immersive technologies offer diminishing returns or justify their complexity and cost for enhancing attention restoration.

### Research hypothesis

Kaplan's Attention Restoration Theory (ART) posits that natural environments are particularly effective at restoring directed attention after mental fatigue. The key mechanism is the promotion of “soft fascination,” which allows for effortless cognitive engagement and recovery. While this effect is well-documented in real-world settings, a crucial question remains regarding its translation into mediated experiences. Different technologies, from traditional screens to immersive VR, vary in their capacity to create a sense of presence and engagement, which may directly influence their restorative potential. This study, therefore, aims to investigate whether the restorative benefits of nature can be elicited through different technological media and, more specifically, to compare the effectiveness of these media in improving attentional performance as measured by the Sustained Attention to Response Task.

Based on traditional mediated Attention Restoration Theory frameworks, we initially hypothesized a linear immersion effect, predicting that attentional restoration would progressively increase with the sensory scope of the medium (Smartphone < Tablet < VR). However, considering the cognitive overhead typically documented in novel virtual environments, we also evaluated an alternative threshold model where extreme immersion (VR) fails to provide a selective cognitive advantage over a moderate, hands-free display (Tablet).

## Materials and methods

### Design

This study employed a mixed experimental design with participants randomly assigned to one of three immersion conditions (in ascending order: smartphone, tablet, or VR headset). The study operationalized attention restoration as the difference in performance on a SART before and after a 5-min nature video. Additionally, participants reported their daily time spent in nature and using their assigned device, as well as their self-reported connection to nature, which were considered as potential moderating variables.

### Participants

A total of 90 participants aged between 17 and 62 years (*M* = 30.2, *SD* = 11.5) were recruited from the University of Toulouse Jean-Jaurès in France. Participants were either contacted by social media or approached directly within the university's buildings and nearby areas by experimenters, without the use of flyers or posters. Participation was voluntary, and individuals were informed that they could withdraw from the study at any time without any penalty. Psychology undergraduates were offered academic credit in the form of a half-point bonus in a course of their choice as compensation for their participation.

Eligibility criteria required participants to be between 17 and 65 years old (encompassing the standard age range of the recruited university undergraduate population), with no uncorrected auditory or visual impairments. Individuals were excluded if they reported a diagnosis of attention disorders such as ADHD, major cognitive impairments, or any condition likely to interfere with attentional performance. Participants were also excluded if they reported any possible dangerous or unpleasant aversions to virtual reality usage, such as a history of epilepsy or a susceptibility to cybersickness.

The study received ethical approval from the ethics committee of the University of Toulouse (Protocol number: 2025_1039). Furthermore, informed consent was explicitly obtained from the legal guardian of the single 17-year-old participant, in addition to the participant's own assent.

### Material

#### Nature video

The natural environment stimulus consisted of a 5-min segment from a YouTube video titled “*Virtual Relaxation 8K 360*° *VR Video—Wenatchee River—Relaxing Birds Songs and River Sounds”* (https://www.youtube.com/watch?v=ofUi0jY–jo). The video depicts a serene riverside scene accompanied by ambient bird songs and flowing water sounds and has a 360° view feature. On the smartphone and tablet, the viewing angle was fixed to straight ahead, whereas in the VR condition, participants could freely navigate the 360° environment through head movements. Across all conditions, audio was delivered via the devices' standard integrated speakers set at a normalized, comfortable volume. The duration of 5 min was selected based on previous findings in the literature, which suggest that a minimum of 5 min of nature exposure is enough to reliably induce measurable improvements in directed attention (Berman et al., 2008; Gamble et al., 2014).

#### Devices

Participants viewed the nature video on one of three types of devices, each corresponding to a different degree of technological immersion. In the smartphone condition, participants used a device with a 5.81-inch screen. The tablet condition included devices with screen sizes between 9.7 and 10.5 inches. For the virtual reality condition, participants used the Oculus Go headset (FOV 100°, resolution 2560 x 1440 pixels).

#### Sustained attention to response task

Attentional recovery was measured using the Sustained Attention to Response Task, a computerized go/no-go task originally developed by Robertson et al. (1997) to assess lapses in sustained attention. The SART is widely considered a gold standard for evaluating directed attention, particularly in the context of ART research, due to its sensitivity to fluctuations in executive attentional control over extended periods. As Berto (2005) states, it is very easy to pick up and very cognitively fatiguing, and even though Berto's study, which used the SART, failed to be replicated by Neilson et al. (2021), its vastly repeated use in ART studies over the last decade supports its validity for detecting improvements in attentional functioning following restorative nature-based interventions.

The task consists of a rapid serial visual presentation of single-digit numbers (1–9) appearing individually on a screen. Participants are instructed to respond to every number (by pressing a key) except for the target number (typically 3), to which they must withhold their response and do nothing. Numbers are presented at a fast and regular pace, requiring continuous attention and inhibitory control to avoid making commission errors. The SART generates two distinct performance indices: commission errors and omission errors. Commission errors occur when participants respond to a target stimulus when they should have withheld their response, reflecting a failure of inhibitory control. In contrast, omission errors represent failures to respond to non-target stimuli and may reflect inattentiveness or deliberate response strategies.

In this study, we had initially planned to only analyze commission errors scores as the primary dependent variable. Our reasoning behind this choice was that (1) commission errors are widely accepted as a direct and reliable indicator of lapses in directed attention, making them particularly relevant for studies grounded in ART, and (2) omission errors can be artificially inflated if participants adopt a “waiting strategy,” deliberately slowing their responses to avoid committing commission errors. Such behavior would violate the task's core instruction to respond “as fast and as accurately as possible” and would distort the assessment of sustained attentional capacity. Furthermore, omission errors have been reported as relatively rare in previous studies, making them less reliable for statistical analysis, and it has been shown that focusing exclusively on commission errors still provides a valid measure of attentional failures (Schepers et al., 2023). However, relying solely on commission errors risks masking deficits in participants who disengage entirely due to severe fatigue. Omission errors signal a state of hypo-arousal or “zoning out” characteristic of deep attentional depletion (Rizzo et al., 2022). Consequently, combining both error types captures the full spectrum of attentional lapses. Therefore, we adopted a global SART error score (sum of commission and omission errors) to capture the full spectrum of attentional lapses. This composite measure is supported by Manly et al. (2002), who demonstrated that SART errors possess high ecological validity, predicting everyday “absent-minded” failures (or “action slips”) regardless of whether they manifest as impulsive responses or failures to respond.

The SART was administered through PsyToolkit (Stoet, 2010, 2017). This version takes approximately 5 min to complete and features 240 digits with 10% targets. Participants first completed a brief pre-training session before their initial SART to familiarize themselves with the task format and minimize learning effects. Each participant performed two full SARTs: one before exposure to the natural environment video and one immediately after. No additional pre-training was given before the post-exposure SART to ensure comparability between the two assessments, and standard SART parameters were used throughout the study.

#### Questionnaires

Participants completed a short questionnaire assessing their preference for nature, consisting of a single item with three response options: (1) “I love nature a lot,” (2) “I moderately like nature,” or (3) “I do not like nature.” In addition, participants reported their daily average time spent using the immersive technology they were assigned to (e.g., daily time spent on tablets for the tablet group, or using VR headsets for the VR group) and their daily time spent in real natural environments. These measures were included to account for potential confounding variables, such as baseline preference for nature or prior exposure to immersive technology, which could influence our results. We collected these measures via simple self-report items.

### Procedure

After providing informed consent, participants completed the aforementioned questionnaires assessing their preference for nature, their daily time spent using their assigned device, and their daily time spent in natural environments. They then completed the first administration of the SART, preceded by a brief pre-training session to minimize learning effects. Participants subsequently watched the 5-min segment of the nature video on their assigned device. During exposure, they were seated comfortably in a controlled laboratory environment with standardized lighting and ambient noise.

Experimenters remained present but outside participants' field of view so as to not distract them. Immediately after the video, participants completed the post-exposure SART without any additional pre-training. At the end of the session, participants were debriefed and thanked for their participation.

### Data analysis

All statistical analyses were conducted using the Jamovi software (version 2.6.26.0).

The primary outcome was the change in sustained attention performance, measured by the SART scores before and after exposure to the nature video. Statistical significance was set at *p* = 0.05 for all analyses.

First, in accordance with Kaplan's Attention Restoration Theory (Kaplan, 1995), our analysis aimed to determine whether exposure to the nature video effectively restored participants' attention. Because the ART framework posits a strict directional hypothesis regarding cognitive recovery, specifically that nature exposure will decrease mental fatigue, we employed one-tailed paired-samples t-tests to evaluate intra-group improvements. According to this theory, such exposure should lead to an improvement in attentional performance, measured here as a decrease in the global SART score (SART pre-exposure vs. SART post-exposure). To test this fundamental hypothesis within each condition, we compared pre- and post-exposure SART scores using paired-samples *t*-tests. This step allowed us to determine whether each level of technological immersion was, in itself, sufficient to produce a measurable restorative effect.

Next, to evaluate whether the effectiveness of the nature video increased with each level of immersion, the initial plan for the statistical analysis was to conduct a two-way repeated measures ANOVA to assess the impact of the Device (Smartphone, Tablet, VR headset) on SART scores over Time (SART pre-exposure vs. SART post-exposure). To evaluate the underlying assumptions for this analysis, we computed a difference score, SART improvement (SART pre—SART post), which captures the intra-individual change and mathematically represents the Time x Device interaction. An assessment of this assumption using the Shapiro-Wilk test revealed a severe violation of normality for the SART improvement scores in the Tablet group (*W* = 0.828, *p* < 0.001). Given this clear violation, a parametric ANOVA could yield unreliable results. Therefore, we proceeded with the robust non-parametric equivalent, the Kruskal-Wallis test comparing improvement scores across the three device conditions.

This test does not assume a normal distribution and is a more robust method for comparing the groups in this context.

While our sample size (*N* = 90) is adequate for standard non-parametric testing, traditional Null Hypothesis Significance Testing (NHST) presents a major epistemological limit: non-significant results (*p* > 0.05) cannot distinguish between a true absence of effect and mere data insensitivity (Dienes, 2014). To overcome this limitation and formally quantify the evidence in favor of either the alternative or the null hypothesis, our frequentist analyses were systematically complemented by Bayesian inferences (Wagenmakers et al., 2018).

Unlike frequentist analyses, which cannot provide evidence in favor of the null hypothesis (*H*_0_), Bayesian inference quantifies the relative predictive performance of competing hypotheses (*H*_1_ vs. *H*_0_) (Dienes, 2014; van de Schoot et al., 2015). This approach is particularly robust for small-sample contexts, enabling a distinction between “data insensitivity” (inconclusive evidence) and a genuine “evidence of absence” (Kruschke, 2014; Zondervan-Zwijnenburg et al., 2019).

We computed Bayes Factors (*BF*_10_) using a default Cauchy prior width (*r* = 0.707) for the effect size under the alternative hypothesis, which is a standard approach in psychological research (Rouder et al., 2009). Following the classification by Lee and Wagenmakers (2013), a *BF*_10_ > 3 indicates moderate evidence for *H*_1_, while a *BF*_10_ < 0.33 (or *BF*_01_ > 3) indicates moderate evidence for *H*_0_. Values between 0.33 and 3 are considered anecdotal or inconclusive. This framework allows for valid probabilistic conclusions regarding the restorative potential of smartphones despite sample size constraints (Wagenmakers et al., 2018).

## Results

Preliminary analyses were conducted to assess the potential influence of confounding variables. Pearson correlations revealed no significant relationships between SART improvement scores and participants' self-reported nature connectedness (*r* = 0.08, 95*% CI* [−0.13, 0.28], *p* = 0.452) or daily usage of their assigned device (*r* = −0.11, 95*% CI* [−0.31, 0.10], *p* = 0.301).While individual differences in biophilic affinity often moderate subjective psychological restoration, our objective behavioral data suggest that the cognitive friction imposed by the device (e.g., active holding or VR overhead) supersedes these individual traits. Consequently, these variables were not included as covariates in the subsequent main analyses.

### Descriptive statistics

A total of 90 participants were included in the final analyses. Participants' ages ranged from 17 to 62 years old (*M* = 30.2, *SD* = 11.5). Group sizes were as follows: VR (*n* = 30), tablet (*n* = 33), and smartphone (*n* = 27).

Table 1 presents a full descriptive overview of SART scores before and after exposure to the nature video across the three device conditions. The improvement score was calculated as the difference between pre- and post-exposure scores (more positive values indicate better attentional restoration).

**Table 1:** Descriptive statistics on pre and post-exposure results (SART between 0 and 25 in this sample).

Statistic	Device	SART pre	SART post	SART improvement
*N*	Smartphone	27	27	27
	Tablet	33	33	33
	VR headset	30	30	30
Mean	Smartphone	10.1	9.15	0.926
	Tablet	9.70	5.70	4.00
	VR headset	10.3	8.10	2.23
Median	Smartphone	8	8	1
	Tablet	10	6	2
	VR headset	10.0	9.00	2.50
Standard deviation	Smartphone	4.76	6.00	4.75
	Tablet	5.55	3.40	4.25
	VR headset	4.91	4.26	4.18
Minimum	Smartphone	2	0	−10
	Tablet	2	0	−1
	VR headset	2	1	−7
Maximum	Smartphone	20	23	11
	Tablet	24	14	18
	VR headset	20	17	11
Shapiro-Wilk *W*	Smartphone	0.957	0.949	0.981
	Tablet	0.937	0.962	0.828
	VR headset	0.968	0.957	0.967
Shapiro-Wilk *p*	Smartphone	0.319	0.198	0.878
	Tablet	0.056	0.289	< 0.001
	VR headset	0.490	0.262	0.466

In the smartphone condition, participants showed a mean pre-exposure SART score of 10.1 (*SD* = 4.76) and a post-exposure score of 9.15 (*SD* = 6.00), corresponding to a mean improvement of 0.926 (*SD* = 4.75). The tablet group improved from 9.70 (*SD* = 5.55) before exposure to 5.70 (*SD* = 3.40) after exposure, yielding the largest average gain of 4.00 (*SD* = 4.25). Finally, participants in the VR condition improved from 10.3 (*SD* = 4.91) before exposure to 8.10 (*SD* = 4.26) after exposure, with an average gain of 2.23 (*SD* = 4.18).

**Figure 1:**
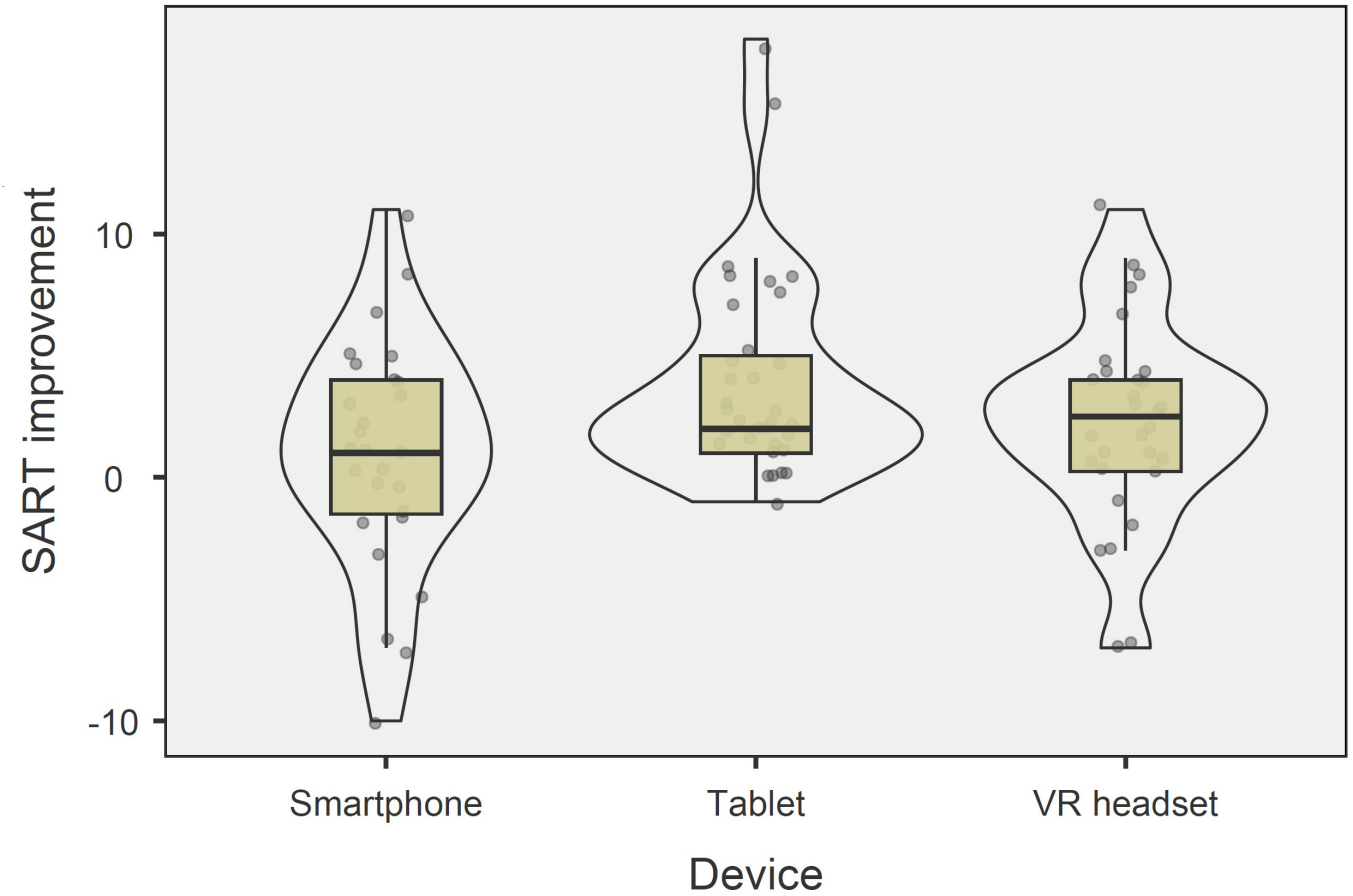
Distribution of SART improvement scores (Pre-exposure minus Post-exposure) across device conditions. Violin plots illustrate the density of the distributions, highlighting the non-normality in the tablet condition. Box plots display the medians and interquartile ranges, while jittered dots represent individual participant scores.

As shown in Figure 1 and the descriptive data, an attentional restoration effect (a positive SART improvement score) is present across all conditions, although its magnitude varies. The mean improvement appears to be greatest for the tablet group, notably higher than both the VR headset and smartphone groups.

### Inferential analysis

#### Measuring attention restoration within devices

To evaluate the restorative efficacy of each specific intervention, a hybrid statistical approach was employed. Prior to conducting the analyses, the assumption of normality for the improvement scores was assessed using the Shapiro-Wilk test. The assumption was met for the Smartphone and VR conditions, allowing for Paired Samples *t*-tests. However, the Tablet condition exhibited a significant deviation from normality (*p* < 0.001), prompting the use of the robust non-parametric Wilcoxon Signed-Rank test for this specific group to prevent inflated Type I errors (Wilcox, 2012).

For the smartphone condition, the frequentist paired *t*-test did not reveal a significant difference in SART scores (*t*(26) = 1.01, *p* = 0.160), and the Bayesian analysis yielded anecdotal evidence (*BF*_10_ = 0.537). According to standard conventions (Lee and Wagenmakers, 2013), this indicates that the data are insufficiently sensitive to definitively confirm or reject the restorative effect of a low-immersion screen. In contrast, the tablet condition yielded a highly significant improvement (Wilcoxon *W* = 432, *p* < 0.001, *r*_*rb*_ = 0.986) supported by extreme Bayesian evidence (*BF*_10_ = 6573). Given the maximum improvement score of 18 in this group, a *post-hoc* sensitivity analysis was conducted excluding this extreme outlier; the intra-group restorative effect for the tablet remained robustly significant (Wilcoxon *W* = 403, *p* < 0.001, *r*_*rb*_ = 0.985), confirming that the effect was not solely driven by extreme values. Finally, the VR headset condition also showed a significant restoration effect (*t*(29) = 2.92, *p* = 0.003, *d* = 0.534) corroborated by strong Bayesian evidence (*BF*_10_ = 12.7).

#### Measuring attention restoration between devices

To examine whether attentional restoration differed across levels of immersion, we conducted a non-parametric ANOVA (Kruskal-Wallis) on SART improvement scores (pre minus post; Manly et al., 2002; Cheyne et al., 2009) with device condition as the grouping variable.

The Kruskal-Wallis test did not reveal a statistically significant difference in SART improvement scores between groups at the conventional alpha level, showing only a marginal trend (χ^2^(2) = 4.94, *p* = 0.085, ϵ^2^ = 0.055). Subsequent pairwise comparisons using the Dwass-Steel-Critchlow-Fligner method reflected this lack of strong inter-group divergence: the difference between the tablet and smartphone conditions failed to reach significance, indicating only a trend (*W* = 3.21, *p* = 0.060). No significant differences were observed between the VR and smartphone conditions (*W* = 1.50, *p* = 0.540), nor between the tablet and VR conditions (*W* = −1.49, *p* = 0.544).

A Bayesian Repeated Measures ANOVA was conducted to rigorously quantify the evidence regarding the interaction between the measurement time and the immersion level. The analysis of effects across matched models van Doorn et al. (2021) revealed extreme evidence for the main effect of the Time condition (*BF*_inclusion_ = 22,999.8), confirming the overall attentional restoration. However, the evidence for the Time × Device interaction was only anecdotal (*BF*_inclusion_ = 2.33). This indicates that the current sample lacks sufficient sensitivity to assert a definitive systemic modulation of the restoration trajectory by the specific device, placing the most probable model as the one including only the main effect of time. *Post-hoc* comparisons between devices provided further nuance in Table 2.

**Table 2:** Bayesian comparison of SART improvement scores between conditions.

Comparison	BF10	Evidence strength
Smartphone vs. tablet	1.203	Anecdotal evidence (inconclusive)
Smartphone vs. VR	0.215	Moderate evidence for H0
Tablet vs. VR	0.764	Anecdotal evidence (inconclusive)

Comparisons are based on non-parametric Bayesian independent samples *t*-tests computed using Jamovi (version 2.6.26.0). Bayes Factors (*BF*_10_) were calculated using a default Cauchy prior width (*r* = 0.707) for the effect size. Interpretation categories follow Lee and Wagenmakers (2013).

As detailed in Table 2, the *post-hoc* comparisons between the devices yielded anecdotal Bayes Factors for the comparisons involving the tablet (Smartphone vs. Tablet, *BF*_10_ = 1.20; Tablet vs. VR, *BF*_10_ = 0.764). This indicates that the current data are insensitive to definitively distinguish the magnitude of the restorative effects when the tablet is directly compared to the other extremes. However, the comparison between the lowest and highest immersion conditions (Smartphone vs. VR headset) yielded moderate evidence in favor of the null hypothesis (*BF*_10_ = 0.215, equivalent to *BF*_01_ = 4.65). This specific finding is theoretically crucial: it indicates that the data are approximately 4.6 times more likely to occur under the assumption of no difference between these two devices. It suggests that the substantial technological overhead and sensory isolation imposed by the VR headset do not translate into a systematically superior cognitive restoration compared to a basic smartphone, reinforcing the hypothesis that high immersion incurs a cognitive cost that offsets the benefits of the nature stimuli.

## Discussion

### Main findings

This study aimed to investigate whether exposure to a natural environment through different devices could consistently restore directed attention, and whether attentional restoration would increase with the degree of immersion each device provides. Building on the theoretical framework of Attention Restoration Theory (Kaplan and Kaplan, 1989), this experiment compared three different levels of technological immersion, each elicited by a different device: smartphone (low immersion), tablet (moderate immersion) and virtual reality headset (high immersion). These three levels of immersion were compared in their ability to provide attentional restoration after viewing a nature video, measured by a performance on the Sustained Attention to Response Task (Robertson et al., 1997).

While participants in the tablet condition exhibited the greatest absolute improvement in sustained attention after viewing the nature video, this difference only approached statistical significance when compared directly to the smartphone condition. The VR headset condition exhibited significant attentional restoration as well, but did not differ significantly from the tablet or the smartphone conditions. Lastly, the smartphone condition did not show a statistically significant attentional restoration. These results confirm that the degree of immersion provided by the technological device used to simulate nature in ART-based interventions is indeed a key factor to take into consideration. However, rather than following the initially posited linear trajectory, these findings strongly support our alternative threshold hypothesis, indicating that intermediate immersion (tablet) optimizes cognitive recovery while avoiding the operational friction of extreme immersion (VR).

### Smartphones' shortcomings

While Ward et al. (2017) proposed the “brain drain” hypothesis, suggesting that the mere presence of a smartphone consumes cognitive resources, a recent meta-analysis and a replication study have challenged the robustness and replicability of this effect (Parry, 2024; Ruiz Pardo and Minda, 2022). A more empirically supported explanation for the lack of restoration in our smartphone condition lies in the biomechanics of “active holding”. Unlike the tablet, which was propped on a stand, or the VR headset, which was strapped to the head, the smartphone required continuous manual stabilization. This difference introduces a methodological confound, as immersion is conflated with posture and interaction mode.

Maintaining static limb positioning engages proprioceptive monitoring and corrective muscle activation, processes that compete for the central executive resources required for cognitive restoration (Canning, 2005; Nagamatsu et al., 2011). This “postural dual-tasking” likely creates a condition of high cognitive load that can interfere with the soft fascination typically elicited by nature stimuli. The Bayesian framework revealed that our data were inconclusive in determining the smartphone's restorative efficacy (*BF*_10_ = 0.537). Rather than confirming a complete failure of the device, this lack of significant improvement points to a potential threshold effect in the Attention Restoration Theory (Kaplan, 1995). The smartphone's restricted field of view and the extraneous cognitive load required for active holding likely create high intra-individual variability: while some users might find soft fascination, for others, environmental distractions overpower it, resulting in an overall insensitive statistical outcome across the sample.

### Virtual reality's shortcomings

The failure of VR to significantly outperform the tablet condition may be attributed to “novelty-induced cognitive load,” often described as the “wow effect”. For participants with limited VR experience, the immediate sensory magnitude of the immersive environment triggers an orienting response that demands significant top-down processing (Makransky et al., 2021; Parong and Mayer, 2021). Instead of inducing “soft fascination,” which allows the directed attention system to rest (Kaplan, 1995), the VR environment likely triggered “hard fascination” or intense curiosity. In this state, the user actively scans the environment to process the technology itself rather than passively receiving the restorative stimuli.

This suggests a non-linear threshold for restorative efficacy: while VR offers a superior “place illusion” (Slater, 2009), the cognitive cost of processing this illusion in a novel context may cancel out the benefits of the natural scenery. Crucially, our Bayesian *post-hoc* comparisons not only showed that data were insensitive to distinguish between the tablet and VR (*BF*_10_ = 0.764), but they also revealed moderate evidence in favor of the null hypothesis when comparing VR to the smartphone (*BF*_10_ = 0.215). This specific finding provides evidence that the substantial technological overhead of the VR headset offset its immersive advantages, resulting in a restorative outcome that is statistically indistinguishable from the lowest-immersion device. This strongly questions the assumption that higher technological immersion automatically translates into superior cognitive restoration.

Furthermore, compatibility issues related to the hardware cannot be overlooked. A sense of “danger” or social discomfort may arise from the loss of visual contact with the real environment, especially for novice users being observed in a laboratory setting (Kaplan, 2001). Technical limitations of the headset, such as blurriness and eye strain (Rebenitsch and Owen, 2016), may have further imposed a physiological friction that hampered the restorative experience.

### Theoretical implications

The reliance on the global SART score proved crucial in capturing the full spectrum of attentional depletion. Specifically, the presence of omission errors in our sample indicates that participants experienced periods of complete attentional disengagement (“zoning out”), a state that commission errors alone fail to capture but which is central to the concept of directed attention fatigue (Cheyne et al., 2009; Manly et al., 2002). By utilizing a hybrid frequentist and Bayesian approach, this study thoroughly quantified the evidence across conditions, revealing that the lack of significant restoration on smartphones stems from data insensitivity (*BF*_10_ = 0.537) rather than a proven null effect. Contrary to the hypothesis that higher immersion linearly increases restoration, our Bayesian Repeated Measures ANOVA showed only anecdotal evidence for an interaction between the device and time (*BF*_inclusion_ = 2.33). Not only did the data fail to support the superiority of the highly immersive VR headset over the moderately immersive tablet, but the pairwise Bayesian analysis also provided moderate evidence (*BF*_10_ = 0.215) that VR confers no restorative advantage over a standard smartphone (Dienes, 2014). This strongly supports the presence of a non-linear immersion threshold, where the cognitive costs of extreme sensory isolation completely counterbalance the benefits of soft fascination (Basu et al., 2019; Makransky et al., 2021). From a practical perspective, this suggests that the additional cost, hardware requirements, and potential physical discomfort (e.g., cybersickness, weight) of VR may not yield proportional cognitive benefits compared to a high-definition tablet. The tablet may represent a “sweet spot” of immersion, sufficient to engage fascination but free from the technological friction that might hamper the restorative experience in VR.

These findings contribute to the refinement of ART in the context of digitally mediated nature experiences. Indeed, our results challenge the assumption that a greater immersion leads to a greater attentional restoration. Instead, it seems that a necessary immersive threshold is needed for attentional restoration to happen, albeit a carefully managed one; a level of stimulation that is rich enough to sustain fascination but not so intense or demanding as to overwhelm attentional resources. Excessive immersion, as in virtual reality, can impose additional cognitive load or discomfort, while minimal immersion, as with smartphones, may fail to evoke sufficient perceptual and psychological engagement.

Compatibility is not to be overlooked as a driver for attentional restoration. Research on urban landscapes has found it to be the most influential attribute for perceived restorative potential (Abkar et al., 2011). It appears to play a central role again in digital ART-based interventions. In the present study, tablets may have represented a particularly compatible medium: sufficiently immersive to support fascination and extent, yet familiar, comfortable and the right amount of immersiveness to allow participants to remain cognitively available for restoration. In contrast, smartphones may have failed to create sufficient psychological distance from everyday demands and virtual reality may have introduced incompatibilities related to novelty, discomfort or self-consciousness.

### Practical implications

Our results point to the tablet as representing an “optimal immersive balance” for everyday restorative interventions. It provides sufficient visual extent to engage soft fascination, reliably producing substantial intra-group cognitive restoration where the smartphone proved highly inconsistent, all while avoiding the physiological friction and cognitive overhead associated with head-mounted displays. This suggests that in the trade-off between immersion and usability, the “transparent” nature of the tablet interface offers the highest compatibility for users seeking rapid cognitive recovery without the need for extensive equipment or habituation. Tablet-based nature interventions could therefore represent a practical, low-cost, and easily deployable solution for promoting cognitive recovery in academic or work settings during short breaks. Conversely, the use of smartphones for restorative purposes should be approached with caution as their limited immersive capacity and strong association with everyday use may limit their effectiveness.

### Limitations and future research directions

Several limitations in this study should be acknowledged. First, while the overall sample size (*N* = 90) was sufficient to detect robust restorative effects in the tablet and VR conditions, as well as moderate evidence for the null hypothesis when comparing the smartphone to the VR headset, the Bayesian evidence regarding the smartphone's intrinsic restorative potential (*BF*_10_ = 0.537) and its direct comparison to the tablet (*BF*_10_ = 1.203) remained anecdotal. This indicates that a considerably larger sample might be required to achieve conclusive sensitivity regarding the highly variable effects inherent to low-immersion screens (Dienes, 2014). Second, immersion and presence were not directly measured using subjective scales, preventing a more precise examination of how participants experienced each device. Furthermore, the absence of an active or passive control group (e.g., viewing an urban environment or resting) represents a major limitation, preventing the definitive attribution of the pre-post improvements to the nature stimulus itself, as practice effects or simple recovery from a seated break cannot be ruled out. Additionally, given the exploratory nature of this study, we did not conduct an a priori power analysis nor pre-register an analysis plan. This population may possess higher baseline cognitive reserves or different technological habits compared to the general public or older adults.

Additionally, while we controlled for immediate practice effects, we did not account for participants‘ pre-existing levels of fatigue prior to entering the laboratory (e.g., post-exam exhaustion vs. well-rested). Variations in initial depletion states could have influenced the potential for restoration, as ART suggests that restoration is most pronounced when directed attention is already fatigued. Finally, the quality of the virtual reality hardware we used in this study was relatively modest, and higher-resolution or more comfortable systems may yield different results. This is especially supported by some participants' complaints. Participants were also overwhelmingly unfamiliar with virtual reality, reporting no prior experience with VR for most.

It would therefore be valuable for future studies to directly compare novice and experienced users of virtual reality, or to incorporate a familiarization phase prior to experimental exposure in order to mitigate novelty-related effects. To rigorously test the “active holding” hypothesis, future studies should isolate the effect of screen size from biomechanical demands. This could be achieved by including a condition where the smartphone is mounted on a stand, similar to the tablet setup, thereby removing the motor task of gripping the device. If restoration occurs in a hands-free smartphone condition, it would confirm that the motor load, rather than the small screen size, is the primary inhibitor of cognitive recovery. Furthermore, by focusing exclusively on directed attention via the SART, this study isolated the cognitive domain of fatigue. To fully address the mechanistic fragmentation between ART and SRT, future research should adopt multi-modal assessments integrating behavioral data with objective physiological markers of emotional modulation, such as Electroencephalography (EEG) or Heart Rate Variability (HRV). Ultimately, carefully disentangling immersion, presence, and device familiarity will be crucial to properly refine the key attribute of compatibility within digital biophilic environments.

## Conclusion

This study investigated whether exposure to digitally mediated natural environments could restore directed attention after a cognitively depleting task, and whether such restoration systematically increased with the degree of technological immersion. Drawing on Attention Restoration Theory (Kaplan and Kaplan, 1989), we compared smartphone, tablet and virtual reality presentations of a natural scene and assessed attentional restoration using the Sustained Attention to Response Task (Robertson et al., 1997).

Results indicate that digitally mediated nature exposure can support attentional restoration, but that this effect does not increase linearly with immersion. While intra-group analyses suggested varying degrees of improvement, our inter-group analyses yielded no conclusive evidence of a systematic moderation by immersion level. Crucially, high immersion via virtual reality yielded no restorative advantage even when directly compared to the lowest immersion smartphone condition. These results challenge the assumption that attentional restoration in digital contexts depends solely on the intensity of technological immersion provided. Instead, devices that are sufficiently immersive to support fascination, extent, and the sense of being away, yet familiar and minimally distracting enough to avoid excessive cognitive load, may offer the greatest restorative potential. This translates clearly into practical perspectives: simple and widely accessible devices may be more effective than costly highly immersive technologies for everyday attentional recovery. However, future research is needed to assess whether or not the cognitive costs associated with initial exposure to virtual reality are mitigated or even reversed should users become more familiar with the technology.

As digital biophilic interventions continue to show benefits, identifying the optimal balance between immersion intensity, familiarity, and usability will be essential for the development of effective, evidence-based interventions. By characterizing the non-linear dose-response relationship of technological immersion, these findings provide a necessary foundation for designing personalized and adaptive biophilic environments tailored to mitigate mental fatigue in highly demanding cognitive settings.

## Data Availability

The datasets generated and analyzed during the current study are available in the Open Science Framework (OSF) repository at https://osf.io/4av2g/files/m6j9k.
